# It’s Not Always Infections When It Comes to Resource-Poor Countries: A Fascinating Case Report

**DOI:** 10.7759/cureus.66469

**Published:** 2024-08-08

**Authors:** Gowri Swaminathan, Arshia Sethi, Santino Patrizi, Ahmed Elhawary, Nuha Al-Howthi, Utsow Saha, Celeste Defillo-Lopez

**Affiliations:** 1 Internal Medicine, Icahn School of Medicine at Mount Sinai, Queens Hospital Center, New York, USA

**Keywords:** cholestatic liver disease, inflammatory bowel disease, liver transplantation, gastrointestinal infections, ulcerative colitis, primary sclerosing cholangitis

## Abstract

A patient’s demographics often guide healthcare providers toward clues to a diagnosis. A recent travel history becomes an essential piece of the puzzle when there is a high suspicion of an infectious cause. When a patient walks into the hospital after having traveled to or from a resource-poor country with systemic afflictions, a physician’s mind quickly jumps to infectious causes, and in most circumstances, it proves to be correct. We report an interesting case of a 28-year-old male from Guatemala who experienced acute gastrointestinal (GI) symptoms. Previous research in this field has shown that patients with inflammatory bowel disease (IBD) are prone to a slew of GI infections. Interestingly, our patient's presenting symptoms were initially attributed to "infections," but a thorough investigation revealed an unexpected twist of events. Our patient presented with multiple GI infections after the usual triggers, which masqueraded the coexistence of underlying primary sclerosing cholangitis and ulcerative colitis for a short course but were diagnosed promptly after a thorough workup.

## Introduction

The association between inflammatory bowel disease (IBD) and primary sclerosing cholangitis (PSC) is a well-established tenet in medical literature. During the International Liver Congress 2016 in Berlin, Munster et al. [1] noted that PSC was declared one of the most significant unmet needs in hepatology. The gut and liver have been reported to have reciprocal interactions, and it is crucial to identify patients with this high-risk association to facilitate the implementation of appropriate surveillance and monitoring strategies for malignancies [2]. Of all the patients with IBD, 0.8-8% with ulcerative colitis (UC) and 0.4-9% patients with Crohn’s disease (CD) will develop PSC [3]. Irving et al. conducted a cohort analysis of 18,829 patients with IBD, and they found that the risks of common infections, viral infections, and gastrointestinal (GI) infections were increased for people with both UC and CD [4]. We present a unique case of a 28-year-old man from a developing nation who initially presented with multiple GI infections but was eventually diagnosed with PSC and UC.

## Case presentation

A 28-year-old male who had recently moved from Guatemala presented to the emergency room with complaints of non-bloody diarrhea of five days duration. The patient had tried antacids with no relief; he was also experiencing generalized malaise at the time of presentation. He confirmed eating a stale sandwich before developing the symptoms mentioned above. Per the patient, the stool was initially green but then changed to dark brown/black. He also revealed that he had had a prior episode of bright red blood in his stools two years back and required a blood transfusion at the time. The patient had a chronic alcohol dependence history for many years up until four months before the current presentation, and his history was also significant for a cholecystectomy done eight years back. The examination findings were positive for pallor and scleral icterus. Lab tests revealed a hemoglobin of 5.9 g/dl (14-18 g/dL) and thrombocytopenia of 17 x 10^3^/microL (150-450 x 10^3^/microL). His liver function panel showed a cholestatic pattern with alkaline phosphatase of 1092 U/L (40-129 U/L) with a direct bilirubin of 1.9 mg/dl (0.0-0.3 mg/dl), which worsened to 1514 U/l (40-129 U/L) and 2.8 mg/dl (0.0-0.3 mg/dl), respectively, during the hospital stay. After transfusion with pRBCs, his hemoglobin improved. The patient became febrile on the day after admission and was treated with a course of oseltamivir after testing positive for influenza B infection, with autoimmune thrombocytopenia being high on the list of differential diagnoses.

CT of the abdomen with pelvis demonstrated small bowel ileus with dilation but did not show any evidence of cirrhosis. The magnetic resonance cholangiopancreatography (MRCP) did not reveal choledocholithiasis or significant intrahepatic or extrahepatic biliary ductal dilatation. Ultrasonography of the right upper quadrant did not reveal any acute findings either. The autoimmune workup was noncontributory, including antinuclear antibodies, anti-mitochondrial antibodies, and anti-smooth muscle antibodies sent during the hospital stay. He was treated with parenteral iron for severe iron deficiency. Thrombocytopenia, which was most likely due to immune thrombocytopenia and past chronic alcohol use, improved over the hospital stay. However, the patient continued having bloody stools, and the stool polymerase chain reaction (PCR) returned positive for *Campylobacter jejuni*. Blood cultures returned positive for *Klebsiella pneumonia*, and he was treated with ceftriaxone and azithromycin. He was also found to be positive for *Helicobacter* *pylori *infection, for which he was started on the triple therapy regimen of clarithromycin, amoxicillin, and pantoprazole after discontinuing azithromycin. He was discharged from the hospital after clinical improvement. 

**Figure 1:**
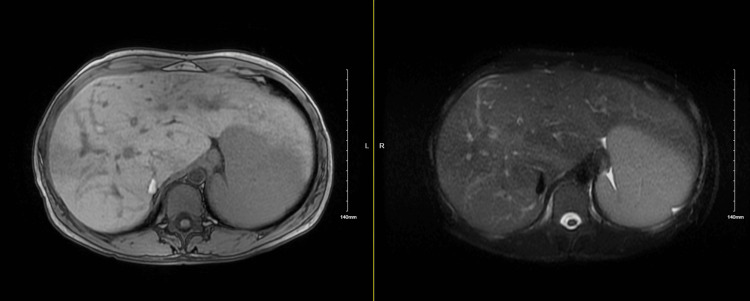
Repeat magnetic resonance cholangiopancreatography (MRCP) with and without contrast, showing significant dilatation of the biliary tree (left: 2D in/out phase, right:T2 fat saturation)

**Figure 2:**
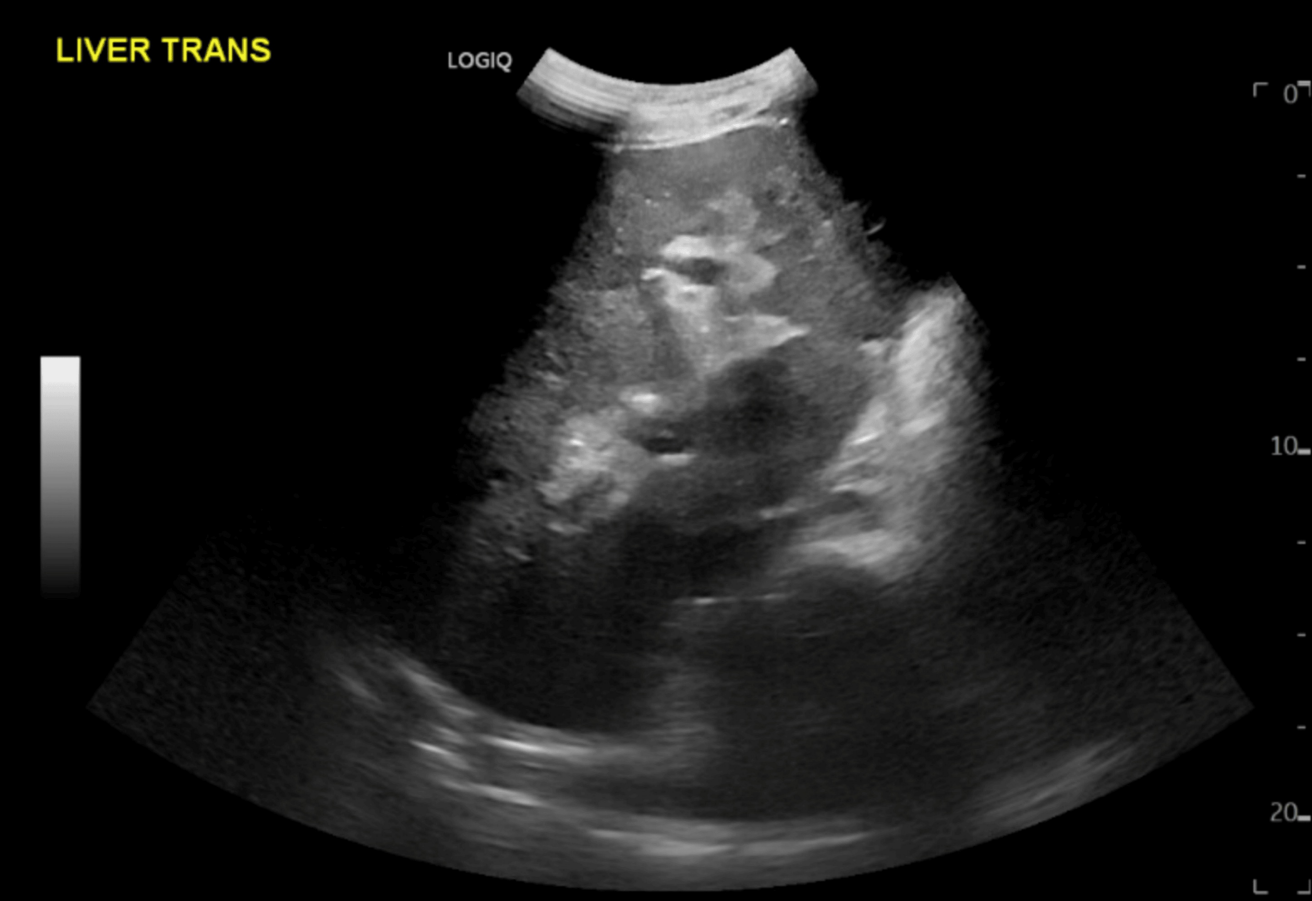
Altered hepatic architecture as seen on the repeat abdominal ultrasonography

**Figure 3:**
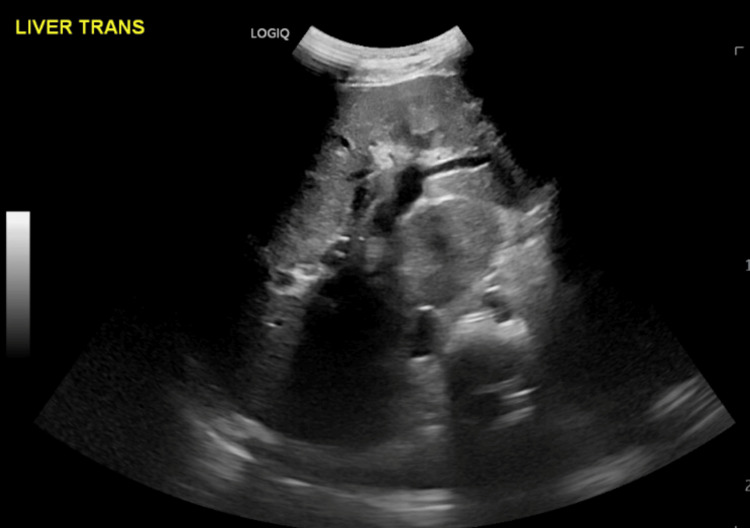
Ultrasonographic evidence of biliary tree dilatation, indicative of primary sclerosing cholangitis

The patient complained of bothersome generalized pruritus at the primary care provider at the two-week follow-up after discharge. He continued to have persistent pruritus and jaundice and was referred for a follow-up at the gastroenterology clinic. He underwent repeat MRCP (Figure 1) about two months after the initial presentation after the abdominal ultrasonography (Figures 2, 3) showed moderate hepatosplenomegaly with coarse hepatic echotexture with the dilated biliary duct. He underwent an IR-guided liver biopsy soon after, which demonstrated extensive sclerosing fibrosis of portal tracts, with marked chronic inflammation, bile duct injury, and cholestasis, along with the presence of scattered foci of hepatocytic necro-inflammation at the border of sclerosing fibrosis, confirming the diagnosis of PSC. The patient was started on ursodiol, which helped relieve his pruritus to a large extent. He also had an elevated IgG level of 4,942 mg/dl (610-1,660 mg/dl), with an increased IgG4 subset of 260 mg/dl (1-123 mg/dl). He was positive for p-anti-neutrophil cytoplasmic antibody (ANCA) and anti-*Saccharomyces cerevisiae* antibody (ASCA). An esophagogastroduodenoscopy was performed, which was not significant; however, the colonoscopy revealed diffuse mucosal changes in the entire examined colon, which was biopsied. The surgical pathology of the specimens was positive for chronic active pancolitis, and the patient was started on mesalamine for ulcerative colitis. He was recently evaluated and accepted by the liver transplant team at a tertiary center.

## Discussion

Our presented case gave us an excellent opportunity to learn the value of perseverant investigation when the index of suspicion of an underlying disease is high despite distractions from other masquerading conditions. The fact that the patient is from a resource-poor country is an important detail that can be a significant distraction, as any infection in the setting of extensive recent travel history can confuse the presenting clinical picture for a healthcare provider. This essentially does not allow for the initial plausibility of underlying chronic diseases as a top differential diagnosis, thus leading to delays in the time to diagnosis. Our patient, upon thorough investigation, was diagnosed with PSC and UC and is currently on the liver transplant list.

GI infections, along with other illnesses like upper respiratory tract infections, acute bronchitis, skin infections, herpes zoster, urinary tract infections (UC only), and pneumonia (CD only), can frequently complicate the clinical course in patients with IBD. In the matched cohort analysis conducted by Irving PM et al. [4], an increase in GI infections was reported in both UC and CD. A sub-analysis of stool culture-confirmed diagnoses of infection with *Salmonella*, *Shigella*, *Campylobacter*, or *Clostridium difficile* by the researchers confirmed that the results were not confounded by the fact that an active IBD can be mistaken for a GI infection [4]. The risk factors that result in an increased incidence of infections in UC and CD, after adjustment for sociodemographic characteristics, comorbidities, and baseline medication use, are malnutrition, surgery, increased pathogen exposure, penetrating disease in CD, low vaccine uptake, impaired response to the vaccines, and glucocorticoid therapy [5,6]. This relates well to our patient from Guatemala, who tested positive for *Campylobacter* and influenza B infection and presented with bacteremia due to *K. pneumoniae* with other pertinent positives of malnutrition, increased pathogen exposure, and no vaccine history. We present this case to underscore the importance of meticulous and diligent investigation in a patient from a resource-poor country with “infectious” symptoms and how it can impact the clinical trajectory.

Several rumors and hypotheses surrounded the most famous composer of all time, Ludwig van Beethoven’s death in 1827, and most of them attributed the tragedy to alcohol-related liver disease. However, it was in 2005 that Karmody and Bachor [7] postulated that Beethoven most likely had had longstanding UC that was complicated by several extra-intestinal manifestations, including PSC and a rare form of sensorineural hearing loss. This is another excellent example of how to avoid the pitfall of pinning the blame on low-hanging fruit, i.e., alcohol in Beethoven's case. It has been debated in the past if PSC and inflammatory bowel disease (IBD) are two distinct entities that happen to share a common susceptibility due to the phenotype or if PSC is merely an extra-intestinal manifestation of IBD [1]. Our patient was diagnosed with PSC first and was found to have UC upon further investigation.

PSC is a rare cholestatic hepatic disease commonly of unknown etiology. It is characterized by chronic inflammation of the bile ducts, which leads to progressive destruction of the intrahepatic and extrahepatic biliary tree. It has a low prevalence ranging between <1 and 32 per 100,000, with the highest incidence found in Northern Europe and North America [8-10] and the lowest incidence in Asia with <1 per 100,000 [8,9,11-13]. PSC has been reported to be infrequent in patients with IBD, with a prevalence of 2-14% of patients in UC [1]; however, on the contrary, 60-80% of patients with PSC have concomitant IBD in Northern Europe and the US [14,15]. PSC has a known propensity to affect males [8], whereas IBD predominantly affects females with a median age of 33 years [16]. This compares well to our presented case, a 28-year-old male at the time of diagnosis. 

UC has been reported to be the more common IBD diagnosis in PSC [17]. However, PSC-IBD is a specific clinical phenotype [15,18-21] with a disease pattern that does not conform to the rules of UC, i.e., typical involvement of the rectum and the contiguous portion of the colon. In this phenotype, high rates of pancolitis (68-83%) and low rates of proctitis (2-4%) have been reported. This is similar to our patient, who had the surgical pathology confirmation of chronic active pancolitis rather than involvement of the descending colon alone. The PSC-IBD phenotype can also be characterized by a phenomenon called backwash ileitis, which occurs due to the impaired ileocecal valve from local inflammation [22]. Our presented case did not reveal this pathology.

Considering the high risk of both colorectal cancer (CRC) and hepatobiliary cancers in this subset of the population, it is imperative that the patients be screened regularly. The risk of CRC is the highest in patients with pancolitis and is further increased with higher degrees of endoscopic and histological inflammation [23,24]. The literature describes PSC subtypes as having elevated serum and/or tissue IgG4 subclass, with a more aggressive disease course than the PSC with normal IgG4. Only about 10-22% of the patients with PSC have high serum concentrations of IgG4 without any evidence of tissue infiltration of IgG4+ lymphocytic infiltration [25]. This cohort of patients represents a poor prognosis [26] and is well comparable to our presented patient, who deteriorated rapidly and is currently waiting for a liver transplant.

Diagnostic modalities used for PSC are MRCP and confirmation with tissue diagnosis by core needle biopsy of the liver. Autoimmune markers that help corroborate clinical findings are antineutrophil cytoplasmic antibodies (ANCA), anti-*Saccharomyces cerevisiae *antibodies (ASCA), immunoglobulin G (IgG), and its subclasses. UC is also confirmed with colonoscopy and tissue diagnosis. Ursodeoxycholic acid and steroids help alleviate the symptoms of pruritus and help with partial recovery; however, systemic steroids come with their share of adverse effects. A liver transplant is the only definitive treatment.

## Conclusions

This case report underscores the importance of diligent investigation and unearthing the actual diagnosis when there are many distractions in a case presentation, such as sociodemographic factors, the acuity of symptoms, and lifestyle choices. It is a well-known fact that patients with PSC-IBD do not have good clinical outcomes due to multiple factors like delays in diagnosis and non-availability of organ donation at smaller centers, which causes significant delays in appropriate treatment. More studies are needed to identify high-risk cohorts within the PSC-IBD phenotype and prognosticate their respective clinical arcs sooner rather than later.
